# Correction: Caregivers of children with HIV in Botswana prefer monthly IV Broadly Neutralizing Antibodies (bNAbs) to daily oral ART

**DOI:** 10.1371/journal.pone.0307065

**Published:** 2024-07-10

**Authors:** Maureen Sakoi-Mosetlhi, Gbolahan Ajibola, Roxanna Haghighat, Oganne Batlang, Kenneth Maswabi, Molly Pretorius-Holme, Kathleen M. Powis, Shahin Lockman, Joseph Makhema, Mathias Litcherfeld, Daniel R. Kuritzkes, Roger Shapiro

## Notice of republication

An incorrect version of Fig 1 was published in error. This article was republished on June 27, 2024, to correct for this error. Please download this article again to view the correct version.
