# Supplementary material for: Caregivers of children with HIV in Botswana prefer monthly IV Broadly Neutralizing Antibodies (bNAbs) to daily oral ART
Source: PLoS One. 2024 Mar 27;19(3):e0299942. doi: 10.1371/journal.pone.0299942 (PMC10971757; doi:10.1371/journal.pone.0299942)
Supplement: S2 Data — (PDF) [file pone.0299942.s002.pdf]

VARIABLE INFORMATION

Variable	Position	Label	Measurement Level
patid	1	PID	Nominal
instn	2	Enrolment Site	Nominal
Wasinformationregardingacceptabilityobtainedfromtheparticipantsp	3	Was information regarding acceptability obtained from the participant's primary caregiver?(Entry Visit)	Nominal
DidthisprimarycaregiverprovideARTtotheparticipantmostofthetimewh	4	Did this primary caregiver provide ART to the participant most of the time when ART was prescribed? (Entry Visit)	Nominal
Howdifficultdoestheprimarycaregiverthinkitwillbetobringthepartic	5	How difficult does the primary caregiver think it will be to bring the participant to and from the clinic for antibody infusions (including all time spent for the visit and travel to clinic)? (Entry Visit)	Nominal
HowdifficultisitfortheprimarycaregivertogiveARTtothestudypartici	6	How difficult is it for the primary caregiver to give ART to the study participant on most days? (Entry Visit)	Nominal
WhataredifficultiesforthisparticipanttakingARTeachdayaccordingto	7	What are difficulties for this participant taking ART each day according to the primary caregiver? Check ALL that apply. (Entry Visit)	Nominal
Ifthemedicalbenefittotheparticipantisthesamehowwouldtheprimaryca	8	If the medical benefit to the participant is the same, how would the primary caregiver compare monthly infusions to daily ART? (Entry Visit)	Nominal
Whatwouldmaketheprimarycaregivermorelikelytochoosetotreattheirch	9	What would make the primary caregiver more likely to choose to treat their child with infusions instead of daily ART? (Entry Visit)	Nominal
IfOtherspecifyEntryVisit	10	If Other, specify (Entry Visit)	Nominal
Whatisthelikelihoodthattheprimarycaregiverwouldrecommendthisstud	11	What is the likelihood that the primary caregiver would recommend this study to someone they know who might be eligible to participate? (Entry Visit)	Nominal
Pleaseprovideothercommentsaboutpositiveornegativeexperiencesrega	12	Please provide other comments about positive or negative experiences regarding the participant's antibody infusions at this clinic. (Entry Visit)	Nominal
Wasinformationregardingacceptabilityobtainedfromtheparticipant_A	13	Was information regarding acceptability obtained from the participant's primary caregiver?(bNAb entry Visit)	Nominal
DidthisprimarycaregiverprovideARTtotheparticipantmostofthetime_A	14	Did this primary caregiver provide ART to the participant most of the time when ART was prescribed? (bNAb entry Visit)	Nominal
Ifthemedicalbenefittotheparticipantisthesamehowwouldtheprimary_A	15	If the medical benefit to the participant is the same, how would the primary caregiver compare monthly infusions to daily ART? (bNAb entry Visit)	Nominal
Whatwouldmaketheprimarycaregivermorelikelytochoosetotreattheir_A	16	What would make the primary caregiver more likely to choose to treat their child with infusions instead of daily ART? (bNAb entry Visit)	Nominal
IfOtherspecifybNAbentryVisit	17	If Other, specify (bNAb entry Visit)	Nominal
Whatisthelikelihoodthattheprimarycaregiverwouldrecommendthisst_A	18	What is the likelihood that the primary caregiver would recommend this study to someone they know who might be eligible to participate? (bNAb entry Visit)	Nominal
Pleaseprovideothercommentsaboutpositiveornegativeexperiencesre_A	19	Please provide other comments about positive or negative experiences regarding the participant's antibody infusions at this clinic. (bNAb entry Visit)	Nominal
Wasinformationregardingacceptabilityobtainedfromtheparticipant_B	20	Was information regarding acceptability obtained from the participant's primary caregiver?(bNAb exit Visit)	Nominal
DidthisprimarycaregiverprovideARTtotheparticipantmostofthetime_B	21	Did this primary caregiver provide ART to the participant most of the time when ART was prescribed? (bNAb exit Visit)	Nominal
Ifthemedicalbenefittotheparticipantisthesamehowwouldtheprimary_B	22	If the medical benefit to the participant is the same, how would the primary caregiver compare monthly infusions to daily ART? (bNAb exit Visit)	Nominal
Whatwouldmaketheprimarycaregivermorelikelytochoosetotreattheir_B	23	What would make the primary caregiver more likely to choose to treat their child with infusions instead of daily ART? (bNAb exit Visit)	Nominal
IfOtherspecifybNAbexitVisit	24	If Other, specify (bNAb exit Visit)	Nominal
Whatisthelikelihoodthattheprimarycaregiverwouldrecommendthisst_B	25	What is the likelihood that the primary caregiver would recommend this study to someone they know who might be eligible to participate? (bNAb exit Visit)	Nominal
Pleaseprovideothercommentsaboutpositiveornegativeexperiencesre_B	26	Please provide other comments about positive or negative experiences regarding the participant's antibody infusions at this clinic. (bNAb exit Visit)	Nominal

VARIABLE VALUES

Value		Label
Howdifficultdoestheprimarycaregiverthinkitwillbetobringthepartic	1	Not difficult at all
	2	Difficult
HowdifficultisitfortheprimarycaregivertogiveARTtothestudyartici	1	Not difficult at all
	2	Difficult
WhataredifficultiesforthisparticipanttakingARTeachdayaccordingto	0	No difficulties
	1	Child dislikes or refuses medication
	2	Caregiver forgets to give medication
	3	Difficult to give when travelling or away from participants
Ifthemedicalbenefittotheparticipantisthesamehowwouldtheprimaryca	0	No preference
	1	Daily ART much preferred
	2	Monthly infusions a little preferred
	3	Monthly infusions much preferred
	4	Monthly ART a little preferred
Whatwouldmaketheprimarycaregivermorelikelytochoosetotreattheirch	0	If clinics were closer
	1	If infusions were better at supressing the virus than ART
	2	If infusions were every three months instead of every month
	3	If infusions were shorter
	4	If infusions were single injection rather than an IV
	5	Other
	6	Nothing, will always prefer daily ART
Whatisthelikelihoodthattheprimarycaregiverwouldrecommendthisstud	1	Very likely
	2	Very unlikely
	3	Neutral
	4	Somewhat unlikely
	5	Somewhat likely
Ifthemedicalbenefittotheparticipantisthesamehowwouldtheprimary_A	0	No preference
	1	Daily ART much preferred
	2	Monthly infusion a little preferred
	3	Monthly infusion much preferred
	4	Monthly ART a little preferred
Whatwouldmaketheprimarycaregivermorelikelytochoosetotreattheir_A	0	If Clinic were closer
	1	If infusions were better at suppressing virus than ART
	2	If infusions were every 3 months instead of every month
	3	If infusion visits were shorter
	4	If infusions were a single injection rather than an IV
	5	Other
	6	Nothing, will always prefer daily ART
Whatisthelikelihoodthattheprimarycaregiverwouldrecommendthisst_A	1	Very likely
	2	Very unlikely
	3	Neutral
	4	Somewhat unlikely
	5	Somewhat likely

Ifthemedicalbenefittotheparticipantisthesamehowwouldtheprimary_B	0	No preference
	1	Daily ART much preferred
	2	Monthly infusions a little preferred
	3	Monthly infusions much preferred
	4	Daily ART a little preferred
Whatwouldmaketheprimarycaregivermorelikelytochoosetotreattheir_B	0	If clinic were closer
	1	If infusions were better at supressing the virus than ART
	2	If infusions were every 3 months instead of every month
	3	If infusion visits were shorter
	4	If infusion were a single injection rather than an IV
	5	Other
	6	Nothing, will always prefer daily ART
Whatisthelikelihoodthattheprimarycaregiverwouldrecommendthisst_B	1	Very likely
	2	Very unlikely
	3	Neutral
	4	Somewhat unlikely
	5	Somewhat likely
