# Supplementary material for: Caregivers of children with HIV in Botswana prefer monthly IV Broadly Neutralizing Antibodies (bNAbs) to daily oral ART
Source: PLoS One. 2024 Mar 27;19(3):e0299942. doi: 10.1371/journal.pone.0299942 (PMC10971757; doi:10.1371/journal.pone.0299942)
Supplement: Attachment — Submitted filename: PONE-D-23-35663. comments.docx [file pone.0299942.s004.docx]

This is a small study focussing on assessing acceptability of once a month bNAbs as against daily antiretroviral therapy among caregivers of 25 HIV infected children. These children were those who were put on ART very early in life and were virologically suppressed. They also had participated in some of the studies under taken by the investigators. bNAbs are likely to become an important area of work in management of HIV disease. Children depend on their parents, more often mothers or other caregivers to provide antiretroviral therapy everyday. Low adherence to ART is common among children. This study finds that monthly bNAb regimen was more acceptable to caregivers compared to daily ART regimen. The authors mention a limitation that it had more close ended questions. However, there are certain areas which require clarifications;

1. All the children and thus their caregivers included in the study had been participating one or the other study protocols, it reduces generalisability of findings additionally.
2. The responses could have been different if the caregivers were informed about the plasma viral loads on real time basis/ or at least before the post-assessment.
3. A significant proportion of children had to be shifted from bNAbs to antiretrovirals again, it is unclear whether the caregivers were informed of the reasons. It would have helped if their responses would have been recorded then as well. It helps in understanding whether care givers convenience or their concern about viral suppression influenced their post-assessment response. This is partly reflected in responses to a question “if infusions were better at suppressing the virus than ART”.
