# Supplementary material for: Caregivers of children with HIV in Botswana prefer monthly IV Broadly Neutralizing Antibodies (bNAbs) to daily oral ART
Source: PLoS One. 2024 Mar 27;19(3):e0299942. doi: 10.1371/journal.pone.0299942 (PMC10971757; doi:10.1371/journal.pone.0299942)
Supplement: Attachment — Submitted filename: FINALIZED POINT-By-POINT-CLEANED_rs (1).docx [file pone.0299942.s005.docx]

**POINT-BY-POINT RESPONSE TO REVIEWER COMMENTS**

Reviewer #1

1. All the children and thus their caregivers included in the study had been participating one or the other study protocols, it reduces the generalisability of findings additionally.

***Response:*** *We appreciate the reviewer’s concerns and acknowledge that working with established cohorts from previous studies within a research setting could be a potential limitation as it provides less information on the real-life applicability of our intervention. However, as with all caregivers caring for children living with HIV, our participants face similar challenges with daily ART administration and have similar desires for simplified/alternative treatment approaches as we have emphasized in the manuscript (Line 33-39). In addition, any child receiving bNAbs in the foreseeable future will be a similar study setting, and studies such as Tatelo will be the only source of acceptability information to help determine the feasibility of this strategy. Therefore the findings of this study can be useful and applicable to other caregivers who find themselves caring for children living with HIV in Botswana and beyond.*

2. The responses could have been different if the caregivers were informed about the plasma viral loads on real real-time basis/ or at least before the post-assessment.

***Response:*** *We thank the* *reviewer for this comment and confirm that all caregivers were informed of their child’s plasma viral load (near real-time) at every scheduled visit per the study procedures. For those who experienced viral rebound during the bNAb only treatment phase, caregivers were contacted immediately and invited to the study clinic where they were notified of the occurrence of the rebound and the implication to their child’s inability to continue in the bNAb-only phase of the study. Upon this notification, study phase exit visits were conducted including administration of the post-intervention acceptability questionnaire. We have modified the data collection and definitions section of the manuscript to reflect these details in lines 99-102 which now reads “Primary caregivers answered closed-ended questionnaires prior to the first bNAb administration (pre-intervention) and after the completion of the final bNAb administration or at the time of viral rebound for those with HIV RNA >400 copies/mL while on bNAb only (post-intervention).*

3. A significant proportion of children had to be shifted from bNAbs to antiretrovirals again, it is unclear whether the caregivers were informed of the reasons. It would have helped if their responses would have been recorded then as well. It helps in understanding whether caregivers convenience or their concern about viral suppression influenced their post-assessment response. This is partly reflected in responses to a question “if infusions were better at suppressing the virus than ART”.

***Response:*** *All children who rebounded while on the intervention phase of the study (monthly dual bNAbs only) were switched back to ART and monthly dual bNAbs were permanently discontinued. Information on caregiver acceptability was collected and assessed at the end of the intervention phase for those with no viral rebound or at the time of viral rebound for those with HIV RNA >400 copies/mL while on dual bNAb only. As highlighted in 2 above, we have updated the study methods to further clarify that caregivers were informed of their child’s HIV plasma RNA levels at every study visit as well as the reason for the permanent discontinuation of bNAbs where appropriate.*

Reviewer #2

1. Being a close-ended questionnaire-based study, the extent of understanding of the caregivers, about the implications of viral rebound in 14 children who were on dual bNAb, is not addressed. Intervention success was defined as maintenance of HIV RNA <400 copies/ml for 24 weeks while on dual bNAb-only treatment. The viremic status of children who had progressed to viral copies >40 from the time of recruitment would be clear only on further follow-up beyond the course of this study. Whether these caregivers were counselled and informed of the above implications at the pre and post-intervention stages prior to answering the questionnaire is not clear.

***Response:*** *Caregivers were made aware of the possibility of their children rebounding while on dual bNAb-only treatment, and its implications for permanent discontinuation of the bNAbs and re-introduction of daily ART. Caregivers of participants who rebounded were given this information as part of the informed consent process and at the time of viral rebound. All the participants who were taken off the intervention step due to detectable plasma HIV RNA were followed weekly until they attained viral suppression and for up to 24 weeks post bNAbs cessation. We have now reported on the time to re-suppression for all participants who were re-initiated on ART before completing the dual bNAb-only treatment phase due to HIV RNA >400 copies/mL. Lines 147 – 149 of the manuscripts now read “All participants who experienced a viral rebound in the intervention phase of the study were immediately re-initiated on ART and all re-suppressed to levels <40 copies/mL at a median of 4 weeks (range: 1 to 20 weeks)”.*

2. The education status and socioeconomic conditions of the caregivers, and the inclusion of subjects only within the study catchment area for close follow-up for at least 12 weeks, may be confounding factors that limit the observations and need to be addressed in detail as limitations of the study.

***Response:*** *We thank the reviewer for this comment and agree that this could potentially limit the generalizability of our findings. However, Table 1 of the manuscript describes the basic socio-demographics of study participants and shows that participants were enrolled from different geographical locations and socioeconomic backgrounds, reducing the possibility of bias. Because the cohort was created by screening nearly all HIV exposed children in the catchment areas, and these areas covered ~ 30% of all exposed children in Botswana during the initial accrual period, and almost 100% of the children who screened positive enrolled, the demographics of the Tatelo cohort are highly representative of children living with HIV in Botswana.*

3. The sample size is very small and the observations should be described as preliminary findings that need to be validated in a larger cohort.

***Response:*** *We agree with the reviewer on the need for the validation of our findings in other cohorts of early-treated infants globally and have included this in our conclusion. Lines 206 - 208 of our conclusion state the need for further research in a larger and more diverse population as encouraged by the reviewer.*
